# Effects of Cold Rolling on the Microstructure and Corrosion Resistance of the Double-Glow Plasma Ni-Cr Alloying Layer on Q235 Steel

**DOI:** 10.3390/ma15227882

**Published:** 2022-11-08

**Authors:** Xiaolin Zhu, Zhengjun Yao, Xiang Chen, Qiang Yao, Pingze Zhang, Guanxi Huang, Baodong Feng, Xuebin Xu

**Affiliations:** 1 College of Material Science and Technology, Nanjing University of Aeronautics and Astronautics, Nanjing 211106, China; 2 Jiangsu Product Quality Testing & Inspection Institute, Nanjing 210007, China; 3 Jiangsu Zhongxin Pipe Sci-Tec Co., Ltd., Nanjing 211100, China

**Keywords:** double-glow plasma surface alloying, Ni-Cr alloying layer, corrosion, cold rolling

## Abstract

A Ni-Cr alloyed layer was prepared on the surface of Q235 steel using double-glow plasma surface alloying (DGPSA) technology and the alloyed layer was cold-rolled with different deformation rates. The microstructure, composition distribution and phase composition of the alloyed layer were characterized using a scanning electron microscope (SEM), an energy dispersive spectrometer (EDS), X-ray diffraction (XRD) and an electrochemical workstation. On this basis, the corrosion resistance of the alloyed layer was analyzed. The results showed that a Ni-Cr alloyed layer formed on the surface of Q235 steel following double-glow plasma nickel–chromium alloying. The alloy elements of Ni and Cr were distributed in a gradient from the outside to the inside and the main phases were FeCr_0.29_Ni_0.16_C_0.06_, Cr_23_C_6_ and γ solid solution. The alloyed layer, once cold-rolled with different deformation rates, underwent synchronous plastic deformation with the substrate, with no fracturing and spalling. The self-corrosion potential of the cold-rolled specimens in 5% H_2_SO_4_ and 3.5% NaCl solution is close to that of 304L stainless steel, and the corrosion currents are much lower. The corrosion resistance of the cold-rolled specimens is comparable to the original specimens, with no significant changes.

## 1. Introduction

**Figure 1:**
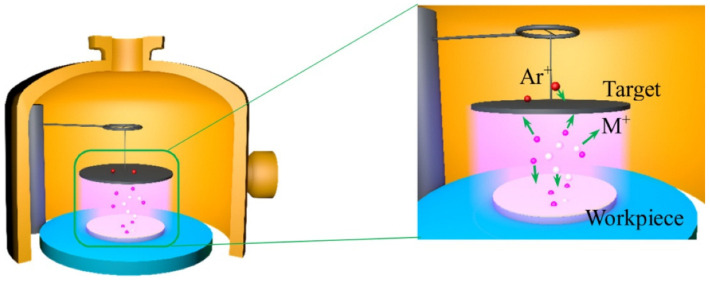
Schematic diagram of the DGPSA technology [1].

DGPSA technology is a surface modification method [1], in which a low-temperature plasma generated by glow discharge excites target material containing alloy elements, evaporated and deposited on the substrate to form a surface-modified layer [2,3]. This modified layer can change the chemical composition and microstructure of the surface/near surface, and improve the ability of the matrix material to resist external damage [4,5,6]. Dual-glow technology originates from the metal element sputtering in the plasma nitriding and sputtering technology, the equipment for which includes a complete vacuum chamber, source electrodes (targets, consisting of one or more required alloy elements) and objects (workpieces), as shown in Figure 1. In the vacuum chamber, the object and source electrode act as two negatively charged components, and the anode is a grounded bell-shaped jar. In the argon plasma atmosphere, when two power sources are connected and reach a certain voltage value, glow discharge occurs at both the cathode and the source. This is the so-called “double-glow” discharge, that is, one glow discharge will heat an object to the alloying temperature and the other glow discharge will bombard the target to sputter the required element. Under the action of an electric field force, these atoms and charged ions from the source electrode will migrate to the surface of the work piece, and then deposited on the workpiece under the action of thermal diffusion, forming an alloying surface [1,7].

The preparation of coatings is one of the ways of improving the properties of the substrate material. After the preparation of coatings, some components can be used directly, while some parts need subsequent cold working. The properties of the material after cold working are directly related to the serviceability of the component. Therefore, it is necessary to study the changes in the microstructure and properties of the coatings after cold working. He [8] et al. investigated the corrosion resistance of a Ni-Co alloy layer prepared by electrodeposition after cold rolling and found that the corrosion resistance of the layer was improved when the cold-rolling deformation was 2–4%, while the deformation over 4% decreased the corrosion resistance. Guo [9] et al. studied the effect of the cold-rolling processing of a Ti-6Al-3Nb-2Zr-1Mo alloy on its corrosion resistance; the results showed that the corrosion resistance increased with the increase of cold-rolling deformation due to the alloy texture and substructure. Wang [10] found that cold-rolling treatments improve the tensile strength and elongation and reduce the elastic modulus of the Ti35Nb2Ta3Zr alloy. Liu [11] investigated that cold- rolled metastable Cr-Mn-Ni-N austenitic stainless steel has higher anodic active dissolution current density, higher passivation current density and lower pitting potential in the electrochemical corrosion.

Double-glow technology can prepare metal or ceramic coatings rich in different elements on the surfaces of steel, copper, titanium and similar material [12,13,14]. This coating is metallurgically bonded to the substrate and has a gradient distribution of chemical composition, and has special properties such as corrosion resistance and wear resistance [15,16,17,18]. However, there are few studies on the changes of the microstructures and properties of the coatings prepared by DGPSA after cold working. In this paper, a Ni-Cr alloyed layer is prepared on a Q235 steel surface using DGPSA technology, and the alloyed layer is cold-rolled with different deformation rates; the microstructure, composition distribution and phase composition of alloyed layer are characterized, and the effect of cold rolling on the corrosion resistance of the alloyed layer is considered.

## 2. Materials and Methods

### 2.1. Test Materials

Q235 steel sized 50 mm × 50 mm × 5 mm is selected as the matrix material, which, once ground and polished, will be cleaned with acetone to remove oil; the source electrode material is an Ni60Cr40 alloy. The chemical composition of Q235 is given in Table 1.

### 2.2. Test and Inspection Equipment

The Ni-Cr alloyed layer prepared by self-manufactured “double-glow plasma alloying furnace” is adopted and the specimens are rolled with different deformation rates (15%, 25%, 35%, 45%, 55% and 75%) using a self-manufactured rolling mill for the laboratory. In the cold-rolling process, the deformation amount is increased by 5% each time until the final deformation amount. The microstructure of coatings is studied using the Sigma FESEM (ZESS, Berlin, Germany), and the composition distribution of alloyed layer is measured by self-contained EDS after the specimen is corroded with a 4% nitric acid alcohol solution. The phase compositions of the samples were studied using D8 Advance X-ray (Bruker, Bremen, Germany) diffraction with Cu Kα radiation at 40 kV and 20 mA, with a scanning speed of 2°/min, with a range from 30° to 90°.

The corrosion behavior of the alloyed layers was evaluated at 25 °C, in comparison with the substrate and 304L stainless steel, using a Princeton P4000A (AMETEK, Berwyn, PA, USA) type electrochemical workstation at a scanning rate of 0.001 V/s. The tests were conducted in a 5% H_2_SO_4_ solution and a 3.5% NaCl solution. A three-electrode system was applied to determine the polarization curves. The alloyed layer was exposed in the solution as a working electrode, with Pt as the auxiliary electrode and the saturated calomel electrode (SCE) as the reference electrode.

## 3. Result and Analysis

### 3.1. Morphology and Phase Analysis of the Alloyed Layer

**Figure 2:**
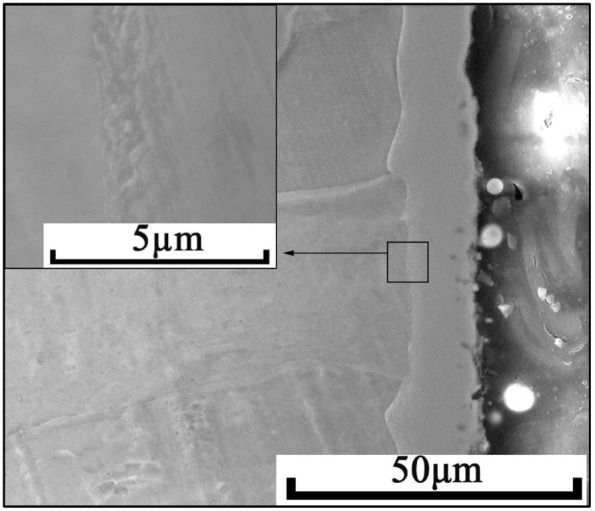
Cross-section morphology of the alloyed layer.

Figure 2 is a SEM photo of the specimen’s cross-section. It can be clearly observed from the figure that an approx. 12 μm thick alloyed layer with a well-defined boundary is formed on the surface of the matrix. This alloyed layer is uniform and compact, with no defects such as voids and cracks, that is to say, the alloy layer is well-bonded with the matrix.

**Figure 3:**
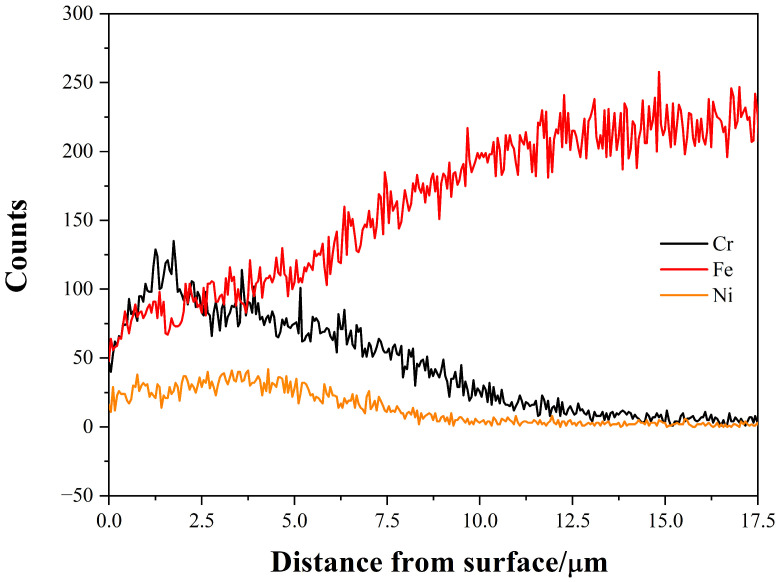
Counts distribution trend of the alloyed elements.

The EDS is used for composition line scanning of the cross-section of the coating along the surface to the matrix. Figure 3 shows the distribution trend of Fe, Ni and Cr. It can be seen from the figure that Ni and Cr reach the peak of Cr 37.35% and Ni 19% (wt%) on the near surface, and then decrease slowly with the increase of distance from the surface. The depletion of the Ni and Cr surface layer may be caused by cathodic glow protection at the initial stage of cooling process, that is, after the source voltage drops to zero, Ar will be continuously introduced while the cathodic voltage is maintained at about 350 V, and the cathodic discharge glow is utilized to prevent the workpiece from contacting an oxidizing substance to prevent oxidation. Under these conditions, the cathode glow discharge temperature is high and there is a positive potential difference of 350 V between the cathode and the anode, so the Ni and Cr on the surface of workpiece are bombarded with ions and re-sputtered; then, atom counter-diffusion occurs on the near-surface region, thus leading to this phenomenon.

**Figure 4:**
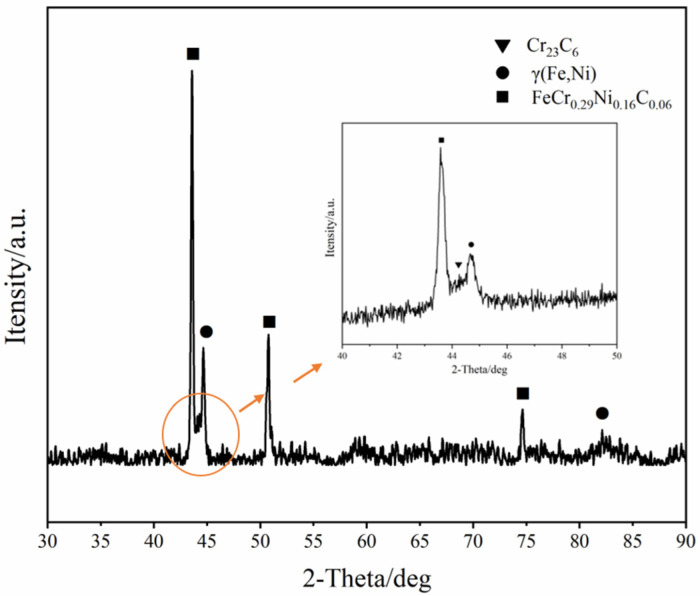
X-ray diffraction pattern of the alloyed layer.

Figure 4 illustrates the XRD pattern of the glow plasma Ni-Cr alloyed layer. It can be seen that the alloyed layer is mainly composed of FeCr_0.29_Ni_0.16_C_0.06_, Cr_23_C_6_ and γ (Fe, Ni) Phase. Ni is a non-carbide-former, so the infiltration of Ni will lead to the inward repulsion of carbon in the surface to form a local C-enrichment region, where C can react with the diffused Cr to form Cr_23_C_6_. The precipitation of hard carbide particles at the grain boundary is highly beneficial to improve the wear resistance of alloyed layer.

### 3.2. Effects of Cold Rolling on the Microstructure of the Alloyed Layer

**Figure 5:**
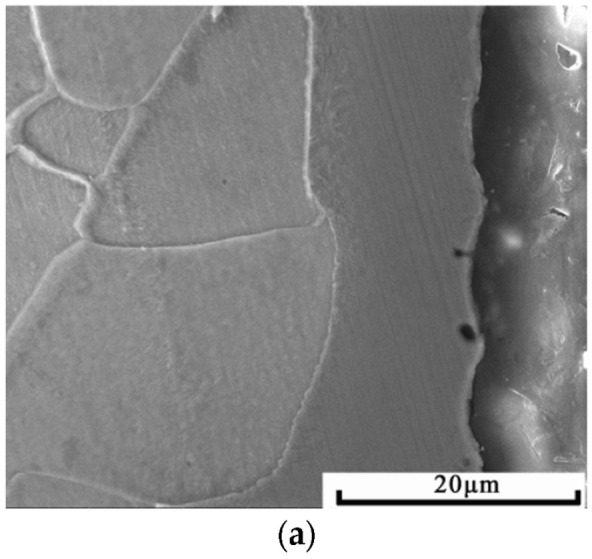

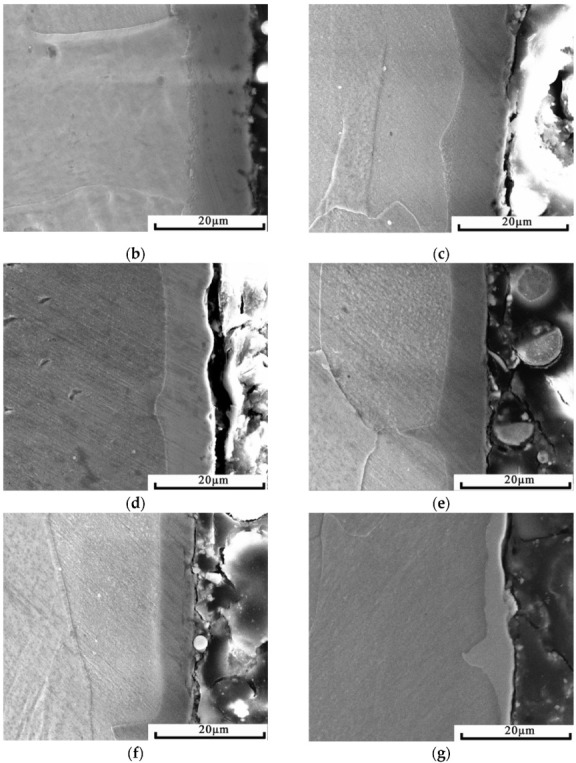
Cross-sectional morphology of each specimen: (**a**) original specimen; (**b**) 15% deformation; (**c**) 25% deformation; (**d**) 35% deformation; (**e**) 45% deformation; (**f**) 55% deformation; (**g**) 75% deformation.

**Figure 6:**
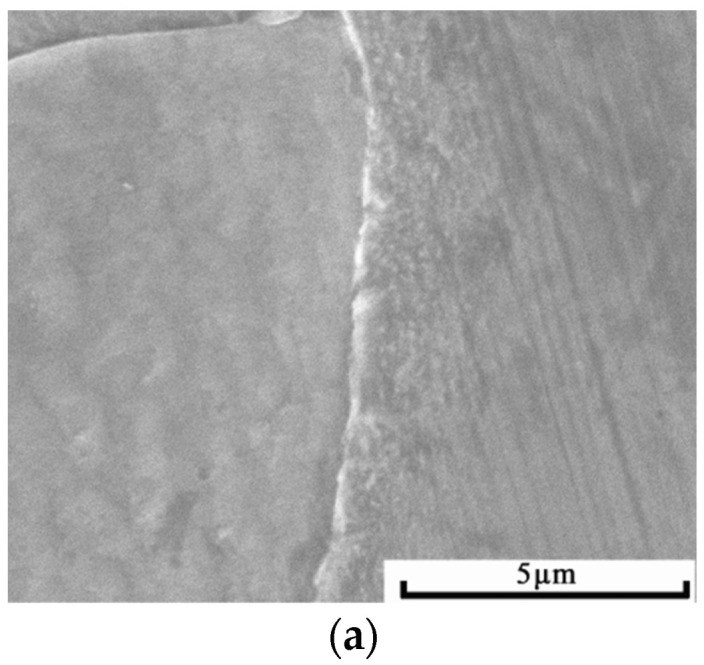

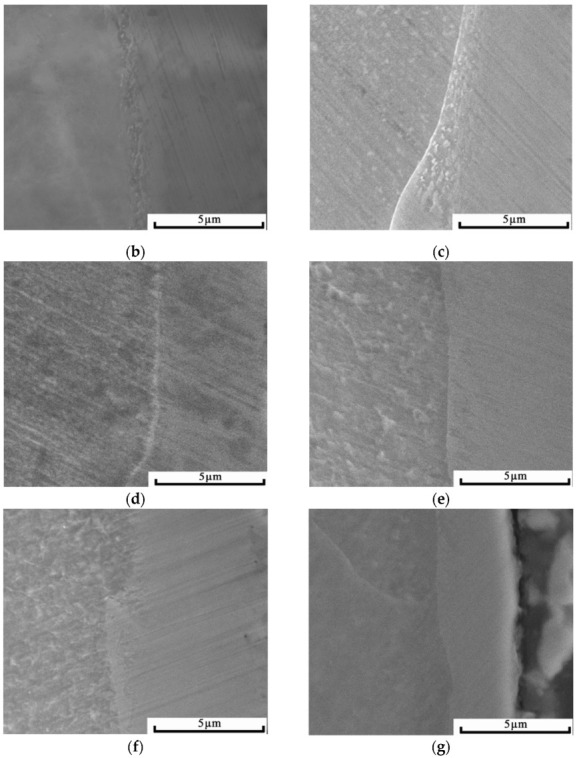
Morphology on the interface between the alloyed layer and matrix of each specimen: (**a**) original specimen; (**b**) 15% deformation; (**c**) 25% deformation; (**d**) 35% deformation; (**e**) 45% deformation; (**f**) 55% deformation; (**g**) 75% deformation.

Figure 5 is an SEM diagram of the cross-sectional morphology of the original specimen through double-glow surface alloying and the specimen cold-rolled with deformation rates of 15%, 25%, 35%, 45%, 55% and 75%, respectively. It can be clearly seen from the figure that the cold-rolled alloyed layer and the matrix have synchronous plastic deformation and the alloyed layer is still homogeneous and pycnomorphic, with no fracturing and spalling. Figure 6 is a partially enlarged view of the interface between the alloyed layer and the matrix of each sample and it can be seen that there is no crack and void on the interface. It is proved that the Ni-Cr alloyed layer prepared by double glow has excellent machinability and good metallurgical bonding with the matrix.

### 3.3. Electrochemical Corrosion Resistance of a Cold-Rolled Specimen in a 3.5% NaCl Solution

**Figure 7:**
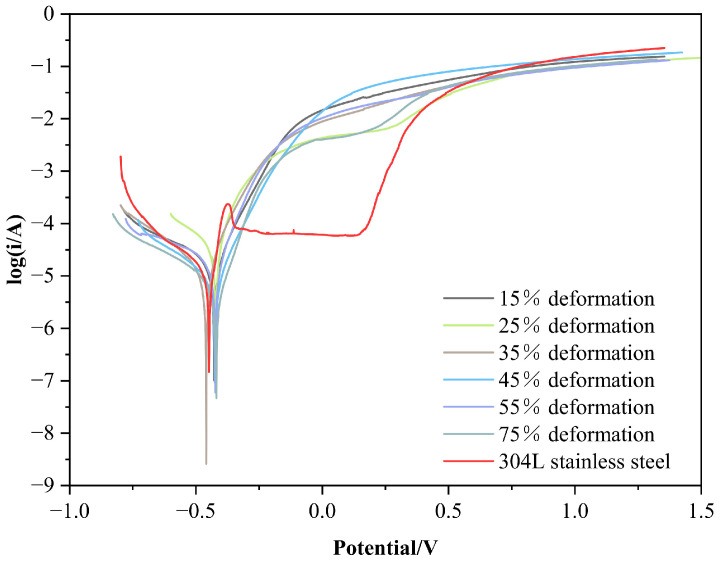
Polarization curves of each specimen in a 3.5% NaCl Solution.

The electrochemical corrosion polarization curves of a cold-rolled specimen with different deformation rates in a 3.5% NaCl solution are shown in Figure 7. Compared with 304L stainless steel, each specimen has no obvious passivation. When the potential value has increased to −0.425 V, the specimen will enter the anodic polarization region and then begin to activate and dissolve, and the corrosion current density will progressively increase with the rise of the potential. Compared with the specimen that has not been cold-rolled, the self-corrosion potential and self-corrosion current density of the cold-rolled specimen and the trend of the corrosion current changing with the potential during corrosion have not changed significantly. This means that different deformation rates have almost no effect on the corrosion resistance of the specimen in this medium.

The kinetic parameters of the electrochemical corrosion of each specimen in the 3.5% NaCl solution are shown in Table 2, from which it can be seen that the self-corrosion potential of the cold-rolled specimen is generally increased by approx. 25 mV compared with that of 304L stainless steel. This means that the tendency of specimens to corrode is reduced in this medium. However, the corrosion current density of the cold-rolled specimen is almost reduced by half, compared with that of stainless steel, so the corrosion rate of the alloyed layer is lower and the corrosion resistance is better. In comparison with the original specimen, the corrosion potential and current density of the cold-rolled alloyed layer have no significant change. This also means that cold rolling with a deformation lower than 75% in a 3.5% NaCl solution has no adverse effect on the corrosion resistance of the alloyed layer.

### 3.4. Electrochemical Corrosion Resistance of Cold-Rolled Specimen in 5% H_2_SO_4_ Solution

**Figure 8:**
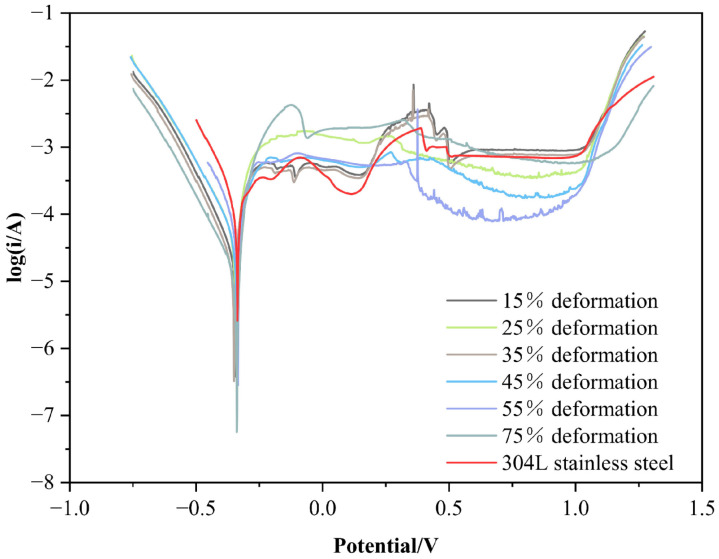
Polarization curve of each specimen in the 5% H_2_SO_4_ solution.

The electrochemical corrosion polarization curves of cold-rolled specimens with different deformation rates in a 5% H_2_SO_4_ solution are shown in Figure 8. Each specimen will enter the anodic polarization region under the potential of approx. −0.345 V, anodic dissolution will occur on the exposed surface and the corrosion current density will increase significantly with the rise of the potential. When the potential has risen to −0.2 V, the corrosion current density will not increase but decrease with the rise of the potential; then, passivation occurs, and the activation–passivation conversion occurs for a time in all specimens; that is, they will not enter the state of stable passivation. The progressive anodic dissolution of the alloyed layer will occur when the potential has risen to approx. 1.5 V and the alloyed layer will enter the trans-passivation region. The passivation potential range of the alloyed layer is much greater and the corrosion characteristics of the alloyed layer are similar to those of the specimen that has not been cold-rolled. This means that the cold-rolling process had no obvious effect on the corrosion resistance of the specimen in the 5% H_2_SO_4_ solution.

The kinetic parameters of the electrochemical corrosion of the cold-rolled specimen, the original specimen and the contrast specimen (304L stainless steel) with deformation rates of 15%, 25%, 35%, 45%, 55%, 75% in a 5% H_2_SO_4_ solution are shown in Table 3. It can be seen from the table that the corrosion potential of cold-rolled specimens is still maintained at approx. −0.345 V, which is equivalent to that of the original specimen and of stainless steel. However, the corrosion current density of each alloyed layer calculated using the Tafel method is in fact a little different from that of the original specimen; the corrosion potential and corrosion current density of the cold-rolled specimen display no obvious change, which means that the corrosion resistance of the specimen cold-rolled in this medium can still reach the standard of stainless steel and that the corrosion resistance of the Ni-Cr layer prepared using double-glow technology will not be damaged by the process of cold rolling.

## 4. Conclusions

(1) A Ni-Cr layer can be prepared on the surface of Q235 steel using DGPSA technology. This alloyed layer is uniform and dense without cracks and holes. Ni and Cr are distributed in gradient from the outside to the inside. The alloyed layer is metallurgically bonded with the matrix and its phase composition is FeCr_0.29_Ni_0.16_C_0.06_, Cr_23_C_6_ compound and γ solid solution.
(2) The Ni-Cr layer cold-rolled with a deformation ranging from 15% to 75% has good plastic toughness and plastic deformation synchronous with the matrix, with no fracturing and spalling.
(3) The self-corrosion potential of the cold-rolled specimens in 5% H_2_SO_4_ and 3.5% NaCl solutions is close to that of 304L stainless steel, and the corrosion currents are much lower. The corrosion resistance of the cold-rolled specimens is comparable to the original specimens, with no significant changes.

## Figures and Tables

**Table 1:** The chemical composition of the Q235 substrate (wt%).

C	Si	Mn	P	S	Cr	Ni	Cu	Fe
0.18	0.13	0.34	0.020	0.012	0.01	0.002	0.01	Bal.

**Table 2:** Electrochemical corrosion test results of different specimens in a 3.5% NaCl Solution.

Test Specimen	Self-Corrosion PotentialE_corr_/V	Self-Corrosion Current Densityi_corr_/mA·cm^−2^	Initiating Passivity Current Densityi/mA·cm^−2^	Maintaining Passivity Current Densityi/mA·cm^−2^	Relative Corrosion Resistance
15%	−0.428	0.0103	-	-	2.65
25%	−0.431	0.0158	-	-	1.73
35%	−0.419	0.0112	-	-	2.44
45%	−0.425	0.0100	-	-	2.73
55%	−0.423	0.0136	-	-	2.01
75%	−0.416	0.0153	-	-	1.78
Original specimen	−0.423	0.0116	-	-	2.35
304L	−0.448	0.0273	0.0631	0.240	1.00

**Table 3:** Electrochemical corrosion test results of different specimens in a 5% H_2_SO_4_solution.

Test Specimen	Self-Corrosion PotentialE_corr_/V	Self-Corrosion Current Densityi_corr_/mA·cm^−2^	Initiating Passivity Current Densityi/mA·cm^−2^	Maintaining Passivity Current Densityi/mA·cm^−2^	Relative Corrosion Resistance
15%	−0.342	0.320	0.575	0.871	1.24
25%	−0.345	0.328	1.480	0.389	1.22
35%	−0.352	0.301	0.562	0.871	1.33
45%	−0.339	0.386	0.676	0.182	1.03
55%	−0.348	0.319	0.589	0.079	1.25
75%	−0.350	0.318	3.980	0.525	1.25
Original specimen	−0.351	0.307	1.510	1.200	1.30
304L	−0.342	0.399	0.363	0.759	1.00
